# Assistive Technology for the Upper Extremities After Stroke: Systematic Review of Users’ Needs

**DOI:** 10.2196/10510

**Published:** 2018-11-29

**Authors:** Anne L van Ommeren, Laura C Smulders, Gerdienke B Prange-Lasonder, Jaap H Buurke, Peter H Veltink, Johan S Rietman

**Affiliations:** 1 Roessingh Research and Development Enschede Netherlands; 2 Biomechanical Engineering University of Twente Enschede Netherlands; 3 Hankamp Rehab Enschede Netherlands; 4 Biomedical Signals and Systems University of Twente Enschede Netherlands

**Keywords:** user perspectives, stroke, upper limb, assistive technology, user-centered design

## Abstract

**Background:**

Technical innovations have the potential to compensate for loss of upper-limb motor functions after stroke. However, majority of the designs do not completely meet the needs and preferences of the end users. User-centered design methods have shown that the attention to user perspectives during development of assistive technology leads to devices that better suit the needs of the users.

**Objective:**

To get more insight into the factors that can bring the design of assistive technology to higher levels of satisfaction and acceptance, studies about user perspectives on assistive technology for the upper limb after stroke are systematically reviewed.

**Methods:**

A database search was conducted in PubMed, EMBASE, CINAHL, PsycINFO, and Scopus from inception to August 2017, supplemented with a search of reference lists. Methodological quality of the included studies was appraised. User perspectives of stroke survivors, carers, and health care professionals were extracted. A total of 35 descriptive themes were identified, from which 5 overarching themes were derived.

**Results:**

In total, 9 studies with information gathered from focus groups, questionnaires, and interviews were included. Barriers and enablers influencing the adoption of assistive technology for the upper limb after stroke emerged within 5 overarching but highly interdependent themes: (1) promoting hand and arm performance; (2) attitude toward technology; (3) decision process; (4) usability; and (5) practical applicability.

**Conclusions:**

Expected use of an assistive technology is facilitated when it has a clear therapeutic base (expected benefit in enhancing function), its users (patients and health care professionals) have a positive attitude toward technology, sufficient information about the assistive technology is available, and usability and practical applicability have been addressed successfully in its design. The interdependency of the identified themes implies that all aspects influencing user perspectives of assistive technology need to be considered when developing assistive technology to enhance its chance of acceptance. The importance of each factor may vary depending on personal factors and the use context, either at home as an assistive aid or for rehabilitation at a clinic.

## Introduction

Stroke is one of the main causes of permanent disability [1,2]. The risk of stroke increases substantially with age as the stroke incidence almost doubles with each decade after the age of 45 years [3]. As a result of the aging population, the number of people older than 65 years in the Netherlands is estimated to almost double (from 2.4 million-4.5 million) between 2008 and 2040 [4]. On the basis of the demographic trends alone, the incidence of stroke will rise in the coming decades. Besides, the number of deaths because of stroke decreased from 153 per 100,000 inhabitants in 2000 to 110 per 100,000 in 2016 [3], and the number of hospitalizations caused by stroke increased from 370 per 100,000 inhabitants in the year 2000 to 482 per 100,000 inhabitants in 2016 [3]. In addition, the stroke mortality rate is likely to decrease because of improvements in acute and long-term care [5]. The rising trend of stroke incidence and hospitalizations will place great strain on national health care services in the future [6].

The cause of stroke is an interrupted blood flow in the brain, either of hemorrhagic or ischemic cause, leading to disturbed generation and integration of neural commands. Depending on the area in which the interruption manifests, resulting impairments vary. Cognitive, emotional, and sensory disorders are often present after a first-time stroke; however, upper extremity weakness or hemiparesis are the most common impairments [7]. With regard to the arm, only 10% to 15% of stroke survivors regain complete functional use during activities of daily living (ADL) within 6 months after stroke, and approximately, another 40% will regain some dexterity in the paretic arm [8]. Recovery of upper extremity function is one of the primary goals of rehabilitation programs. About 40% of occupational therapy is directly targeted at improving ADL [9]. Several studies have shown that focusing on functional activities, with active contribution of the stroke survivor, is vital in stimulating motor recovery after stroke [10-12]. Loss of functional use of the hand and arm causes severe difficulties in personal care activities, especially when those activities involve handling of objects. This limits the independence of stroke survivors and significantly reduces their quality of life [13,14]. By the end of the first year post stroke, an estimated 40% of stroke survivors still need assistance in ADL [10].

Technical innovations, such as assistive technology (AT), provide the opportunity to compensate for loss of motor function by supporting the upper limb during the execution of ADL [13,15]. The definition of ATs used in this study is based on the definition proposed by Demain et al [16] and Hughes et al [5]. Assistive technology is defined as “Electrical or mechanical devices designed to help people recover movement by offering direct assistance to the movement of the upper extremity.” ATs have great potential to assist in promoting intensive use of the arm and hand, without any increase in clinical contact time in the case of a therapeutic application or help from formal or informal carers in case of assistive application. AT can increase the amount of motivational activities that stroke survivors perform, whether it be hobby or gaming activities they enjoy or work and ADL-related tasks that might help them regain a sense of independence. AT can be used both inside and outside the clinic [5,17]. Remarkably, only 25% of the robotic devices for upper extremity rehabilitation have been tested clinically within the stroke population [18], suggesting limited implementation of robotic devices in practice [19]. The complexity of robotic devices and a mismatch between the needs and preferences of the end users and their environment regarding the design of the device are believed to be the main reasons for this low implementation rate [18,19]. This assumption is also expected to be applicable to AT in a more general sense.

User-centered design (UCD) methods have shown that including user perspectives during the design of AT enables development of devices that better suit the needs of the users [20]. The rationale for user involvement during the design process is to design a device that will be usable, comfortable, understandable, and, ultimately, acceptable for the users [21]. Currently, the design of robotic technology for stroke rehabilitation tends to be technology-driven [22]. Although an extensive list of existing technical solutions for physical therapy of the upper limb has been provided [13], few are clinically tested [18]. When AT was tested clinically, devices that were developed according to UCD showed acceptable to promising usability scores, although room for improvement was left, mainly with regard to usability aspects [23,24]. This supports the importance of taking the perspectives of the end users into account during the design and development of AT.

**Figure 1:**
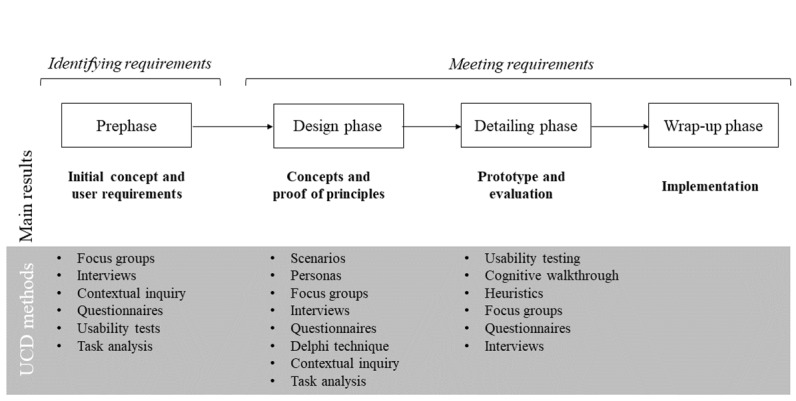
Typical phases of a design project. UCD: user-centered design.

There is a clear need to bring assistive device design to higher levels of acceptance. Ideally, design projects should start with addressing user needs by collecting information about the target population through focus groups, interviews, questionnaires, or observation studies (Figure 1, adapted from Eger et al [25] and Martin et al [26]). Although some studies reported collection of needs and preferences of end users at the start of the design project [5,15,16,19,27-31], the questions asked to gather this information were often too generic.

This study, therefore, systematically reviews existing literature about user perspectives on AT for the upper extremity after stroke. The resulting insights could aid future developers in quickly determining essential user requirements that need to be addressed during the design of AT for the upper extremity after stroke to enhance its chances of acceptance by the users. The insights in this study can thus be used as a starting point for the first phase of AT development, from which developers can proceed to gather more in-depth information from their own use research, specific to their application and intended use. In the later stages of development, it remains important to involve users and incorporate UCD methods (Figure 1) to ensure the device will indeed meet the identified user requirements.

## Methods

### Literature Search

An electronic database search was conducted in PubMed, Scopus, CINAHL, Embase, and PsycINFO from inception to August 2017. The search strategy used in all these databases was a combination of the following keywords and related terms (see Multimedia Appendix 1 for the full syntax):

- Assistive technology, self-help devices, and assistive devices
- Rehabilitation robot, training devices, upper extremity rehabilitation equipment
- Robotics
- Upper extremity
- Cerebrovascular accident and stroke
- User requirements, a priori user perspectives, and patient preferences

Reference lists of potentially relevant papers were scanned to supplement the computerized search results. Furthermore, an internet search (Google Scholar) was performed with regard to factors that affect the use of upper extremity assistive devices in the rehabilitation setting and at home.

### Study Selection

The following criteria were used for the inclusion of studies: (1) studies involving qualitative or quantitative research into user perspectives; (2) involvement of stroke survivors with upper extremity limitations, carers, or health care professionals (HCPs) of stroke survivors; (3) studies concerning upper extremity AT; (4) studies written in English; and (5) published, full-length, and peer-reviewed papers. The definition of ATs used in this review is “Electrical or mechanical devices designed to help people recover movement by offering direct assistance to the movement of the upper extremity,” without distinguishing between devices designed for therapeutic purposes or home use. The included studies needed to comply with all the inclusion criteria. Thus, case studies and studies including user perspectives with regard to a product that will be designed for one specific task were excluded. Moreover, studies evaluating a prototype or product were excluded. After the duplicate citations had been excluded, 2 reviewers (ALvO and GBPL) screened titles and abstracts. Full-text papers were read and summarized independently by 2 reviewers (ALvO and LCS) and discussed subsequently. A final list of papers to be included was created after consensus was reached. A third reviewer could be consulted if there was disagreement between the 2 principal reviewers (JHB in case of titles and abstracts and GBPL in case of full papers).

### Methodological Research Quality Assessment

The Critical Appraisal Skills Program (CASP) checklist was used to appraise the methodological quality of the included studies as it can guide the evaluation of a wide range of methodologies [32,33]. This methodological assessment tool, endorsed by the Cochrane Collaboration, contains 10 items on aims, research design and methodology, participant selection and ethics, data collection and analysis, and the statement of findings, each of which was scored as positive (yes), negative (no), or unclear (cannot tell). Each positive score received 1 point, and each negative or unclear score received 0 points. Thus, the maximum possible methodological quality score was 10. Studies were not excluded based on the CASP score; rather, the CASP score was used as reference to serve as a guide during interpretation of the results.

**Table 1:** Derivation and content of descriptive and analytical themes.

Overarching themes and corresponding descriptive theme		Derived from	Example expressions and citations
**Theme 1: Promoting hand and arm performance**			
	Goal-oriented exercises	[15,19,27-31]	*Therapists stated that training should be oriented at a patient’s goal(s) and his/her ability to accomplish these goal(s).* [29]
	Repetition	[16,28,29,31]	
	Intensity	[16,29,30]	
	Active contribution	[15,19,28,31]	
	Focus on hand and arm	[5,16,27]	
**Theme 2: Attitude toward technology**			
	Motivation	[5,16,27-29,31]	*All participants believed that using home-based technology aimed at arm exercises would help them perform more arm exercises. It will motivate them to engage more in the exercise program*. [27]
	Familiarity and affinity with technology	[28,31]	
	Digital security and privacy	[29,31]	
**Theme 3: Decision process**			
	Knowledge	[5,16,31]	*All patient participants were keen to self-manage. They were all actively engaged in looking for solutions to promote arm recovery and were prepared to spend time and, if necessary, money on potential solutions, including assistive technologies.* [16]
	Evidence-based practice	[5,16]	
	Advice	[5,16,28]	
	Time investment	[16]	
	Safety aspects regulations	[19,27]	
	Trust and expected usefulness	[5,16,27,28,31]	
	Independence and self-management	[5,16,27-30]	
	Money	[5,16,27,30,31]	
**Theme 4: Usability**			
	Donning/doffing	[15,16,19]	*For stroke survivors and families, the devices needed to be easy to get on and off a weak and/or contracted hand/arm...and to be intuitive in terms of correctly positioning the device.* [16]
	Setup	[5,16,27-29,31]	
	Initialization	[15,28,29,31]	
	Portable	[16,27,29,30]	
	Robustness	[5,27,29]	
	Instruction on exercises	[29,31]	
	Comfort	[5,15,19]	
	Lightweight	[15,19]	
	Ease of use	[5,15,16,27-29,31]	
	Compliant	[16,19,27,28,30,31]	
	Adjustment to patient	[16,19,28,29]	
	Technical support	[27]	
	Maintenance	[16,27]	
**Theme 5: Applicability in practice**			
	Monitoring	[15,27,29,30]	*Hardware and software design of technology should facilitate adaptation to individual stroke survivors or patient target groups and to patient progression over time.* [29]
	Feedback	[15,16,28-30]	
	Wrongly executed movements	[29,30]	
	Fatigue and overtraining	[30]	
	Adaptability (patient progression, task setting, and patient group diversity)	[15,19,27-31]	
	Physical comfort	[5,16,19,28,30]	

### Data Extraction

The content of the included studies was analyzed using a structured approach, scanning for information (where available) regarding descriptive features of the population involved and the type of AT and its purpose. Subsequently, factors related to the successful or unsuccessful use of AT were collected and used as input for the analysis of this review. Therefore, information and quotations from participants under the headings *Results* or *Findings* were retrieved from each study.

### Data Synthesis

Meta-synthesis attempts to integrate results from interrelated qualitative studies. In contrast to meta-analysis, meta-synthesis has an interpretive rather than aggregating intent [34]. In this study, the data synthesis was based on the 3-phase process from Thomas and Harden’s thematic synthesis [35]. In the first phase of data synthesis, line-by-line coding of the findings of primary studies was performed by 2 reviewers (ALvO and LCS). Second, descriptive themes based on the expressions found in the first phase were developed. Examples of those descriptive themes can be found in Table 1. Third, the descriptive themes were presented to a multidisciplinary team experienced in the field of rehabilitation technology to develop consensus-based, analytical overarching themes that encompass all descriptive themes. The team consisted of a human movement scientist, electrical engineer, industrial design engineer, biomedical engineer, and a psychologist, of which the majority had not been involved in previous phases of this study. Each study was read several times by 2 reviewers (ALvO and LCS) to ensure that all the perspectives of the participants were captured.

## Results

### Study Selection

**Figure 2:**
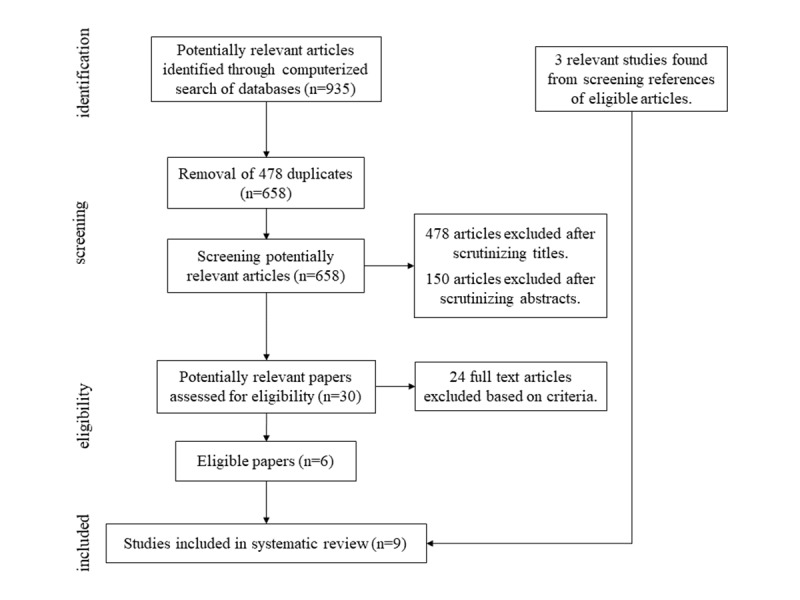
Flowchart of study inclusion.

Initially, 935 references were retrieved from bibliographic databases. After removal of duplicates, 658 potentially relevant papers were screened for retrieval, of which 30 were retained for full-text review. After comparing with the selection criteria, 24 of the full-text papers were excluded. In total, 3 studies were included via additional reference searches of relevant publications. Therefore, the review includes 9 publications. The number of studies included and excluded at various stages of the review process is shown in Figure 2. In all cases, consensus between the 2 raters was reached. Consequently, there was no need to consult the third reviewer.

**Table 2:** Characteristics of included studies.

Source and aim of the paper (N=9)	Target population (number)	Method of data collection	Quality score
Lam et al (2015) [31]; Aim: establish the current use and perceptions of gaming, social media, and robotics technologies for rehabilitative purposes from the perspective of adults with upper-limb impairments to identify barriers and enablers to their adoption and use	Stroke survivors (n=8)	Focus groups	9
Nasr et al (2015) [28]; Aim: examine stroke survivors’ experiences of living with stroke and technology to provide technology developers with insight into values, thoughts, and feelings of the potential users of a to-be-designed robotic technology for home-based rehabilitation of the hand and wrist	Stroke survivors (n=10) and caregivers (n=8)	In-depth interviews	8
Prange et al (2015) [19]; Aim: identify user requirements for development of an active assistive device to support hand opening during functional activities	Stroke survivors (n=5) and HCPs^a^ (n=6)	Interviews	5
Radder et al (2015) [15]; Aim: identify user requirements as input for the development of a wearable soft-robotic assistive device for the support of hand function of elderly and stroke survivors in a wide range of ADL^b^	Stroke survivors (n=4) and HCPs (n=7)	Focus groups	7
Hughes et al (2014) [5]; Aim: understand HCPs’, stroke survivors’, and carers’ experience and views of upper-limb rehabilitation and ATs^c^ to identify barriers and opportunities critical to effective translation of ATs into clinical practice	Stroke survivors and carers (n=79) and HCPs (n=120)	Questionnaire	9
Sivan et al (2014) [27]; Aim: investigate if the ICF^d^ framework is a useful basis to ensure that the key user needs are identified in the development of a home-based arm rehabilitation system for stroke survivors	Stroke survivors (n=9) and HCPs (n=6)	Semistructured interviews	9
Demain et al (2013) [16]; Aim: investigate stroke survivors’, caregivers’, and stroke professionals’ experiences and perceptions of stroke upper-limb rehabilitation and AT use and identify the barriers and facilitators to their use in supporting stroke self-management	Stroke survivors (n=11), family caregivers (n=5), and HCPs (n=6)	Focus groups	8
Hochstenbach-Waelen and Seelen (2012) [29]; Aim: identify criteria and conditions technology should meet to facilitate (implementation of) technology-assisted arm-hand skills training in rehabilitation therapy of stroke survivors	HCPs (n=6)	Semistructured interviews	4
Lu et al (2011) [30]; Aim: discover the needs and preferences of therapists with respect to a robot that focuses on upper-limb rehabilitation	HCPs (n=233)	Questionnaire	9

^a^HCP: health care professional.

^b^ADL: activities of daily living.

^c^AT: assistive technology.

^d^ICF: International Classification of Functioning, Disability and Health.

### Study Characteristics

In total, 9 studies covering 139 stroke survivors and carers and 384 HCPs were included for analysis [5,15,16,19,27-31]. The majority of the studies had at most 20 participants except for 2 studies that applied questionnaires involving over 100 participants [5,30]. The characteristics of the studies are shown in Table 2. All studies described end users' experiences and perspectives regarding the design of AT for use after stroke. In total, 4 studies used interviews [19,27-29], 3 studies used focus groups [15,16,31], and 2 studies questionnaires [5,30] to elicit information from end users.

### Methodological Quality

Quality scores retrieved from the CASP ranged from 4 to 9 points, with 7 studies having a score above 5 out of a possible score of 10 (Table 2). Scores per question of the CASP are shown in Table 3. Studies with lower scores tended to provide insufficient information about particularly the recruitment strategy, the relationship between researcher and participants, the ethical procedures, and the data analysis. A minority of the studies (2/9, approximately 22%) provided information about the role and potential bias of the researcher during the study. Nevertheless, studies with a low quality score were retained for inclusion because of their relevant contribution of data.

**Table 3:** Questions of the Critical Appraisal Skills Program and the number of studies that do or do not comply with each question.

Question	*Yes*	Partially reported or *No*
Was there a clear statement of the aims of the research?	9	—^a^
Is a qualitative methodology appropriate?	9	—
Was the research design appropriate to address the aims of the research?	9	—
Was the recruitment strategy appropriate to the aims of the research?	5	4 [15,19,29,31]
Was the data collected in a way that addressed the research issue?	7	2 [19,29]
Has the relationship between researcher and participants been adequately considered?	2	7 [5,15,16,19,27-29]
Have the ethical issues been taken into consideration?	6	3 [19,29,30]
Was the data analysis sufficiently rigorous?	8	1 [29]
Is there a clear statement of findings?	4	5 [15,16,19,28,29]
How valuable is the research?	9	—

^a^Not applicable.

### Synthesis

**Figure 3:**
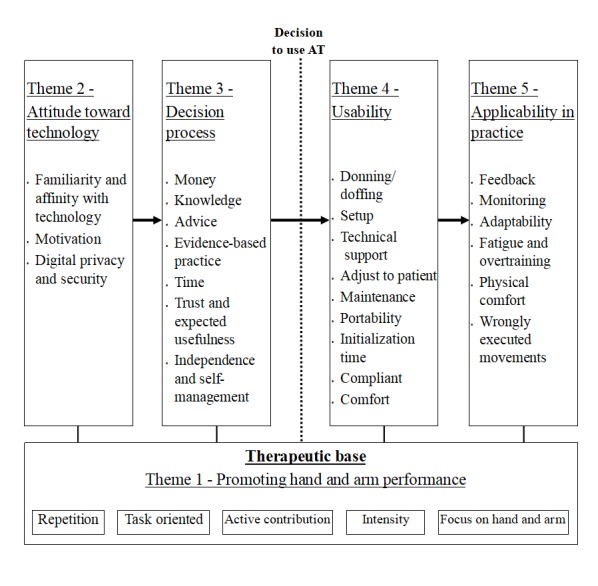
Themes in use and implementation of assistive technology (AT) for the arm and hand according to health care professionals and stroke survivors.

Statements and sentences from primary data were discussed and organized into 35 descriptive themes (Figure 3). On the basis of the descriptive themes, 5 overarching analytical themes were derived: (1) promoting hand and arm performance; (2) attitude toward technology; (3) decision-making process; (4) usability; and (5) applicability in practice, illustrated in Figure 3.

Table 1 provides illustrative quotations from included studies and the corresponding descriptive and overarching themes. During third-stage discussions (analytical theme identification) within the multidisciplinary expert group, underlying relationships between those themes were identified (Figure 3). For an AT to be considered for the support of the upper-limb function in stroke, the device should address a therapeutic base for promoting hand and arm performance (theme 1). A positive attitude toward technology (theme 2) is a prerequisite for starting the decision-making process (theme 3) on whether to use an AT. After it is decided to (consider to) use an AT, aspects determining the usability of the system (theme 4) play a crucial role in the level of user satisfaction. The applicability of an AT in practice (theme 5) depends on factors that may promote long-term use of the device, when properly implemented.

### Theme 1: Promoting Hand and Arm Performance

#### Repetition, Task Oriented, Active Contribution, Intensity, and Focus on Hand and Arm

Therapeutic principles which are the foundation of motoric recovery should be addressed by AT. Stroke survivors and carers have remarked that intensive movement repetition needs to be promoted to regain any degree of function and to optimize recovery [16,28,31]. In their eyes, meaningful movements are preferred during training [28,31] as they want to improve their ability to use their affected limb in functional activities such as combing hair, washing, dressing, cooking, and eating with knife and fork [27]. HCPs in both qualitative [29] and quantitative studies (99%) [30] agree that the intensity and frequency of meaningful task-oriented movements should be enhanced. So training should be tailored to the individual goals, which involves training of the specific task to accomplish the goal, and also comprises components of the tasks that stroke survivors want to remaster [29]. When severely affected, active contribution and training of the severely affected side is preferred, to achieve the ability to use it as supporting hand in bimanual activities [15,19], as is wished by stroke survivors and carers. Tailored to the stroke survivors’ functional level, training should range from gross to fine manipulation and could be provided by games when these are used for rehabilitation purposes [28].

Technology aimed to be used to support the upper extremity should, therefore, offer variability in exercises and its functionality [29]. Computer exercises should enable (virtual) ADL-specific activities through meaningful and functionally relevant activities (88%) [30] based on the principles of motor relearning [27]. Normal movement patterns needed for daily activities, active participation of the hand and arm, and frequent movement repetition should be promoted and trained in the games [28]. Games functionality should be as close as possible to the functionality of real analog games [29].

Over 75% of the stroke survivors, carers, and HCPs mentioned that the current practice in therapy is insufficient [5], as there is therapeutic emphasis on the lower extremity [16,27], whereas additional therapy would enhance their upper extremity functioning [27]. All the end users thought that time efficiency of therapy could be improved with AT allowing additional time for upper extremity training [5].

### Theme 2: Attitude Toward Technology

#### Motivation, Familiarity and Affinity With Technology, and Digital Security and Privacy

Before considering using an AT, the attitude toward technology in general can either play a facilitating role or form a barrier, for both the stroke survivor or end user and the HCP. Beside the before-mentioned factors to promote hand and arm performance, HCPs stress the importance of training to be motivating and challenging for stroke survivors. Motivation of stroke survivors to regain control over movements of their affected side is usually very strong [28]. Control over the affected side can be achieved by dividing large goals into smaller, achievable goals, but it can also be enhanced by including a gaming element in the case of therapeutic devices [29]. Games, either Web-based or offline as AT, are innovative means that can help to motivate stroke survivors to do their therapeutic exercises [28]. Stroke survivors, carers, and HCPs acknowledged the motivational aspect of AT as they were seen as an improvement on routine therapy because they are *high-tech* and more enjoyable [16]. All participants, stroke survivors and HCPs, in the study of Sivan et al [27] thought that using a home-based technology aimed at arm exercises would help them to perform more arm exercises. More independence [27,28] and regaining confidence in their own body are motivating aspects for stroke survivors to engage in the exercise program [28].

However, mixed feelings are expressed about the affinity with technology [28,31]. Feelings about AT are considerably influenced by the familiarity with technology; stroke survivors with technology experience before they suffered the stroke tend to be more positive toward new technologies [28]. Stroke survivors are willing to adopt new technologies if they are proven to be effective; however, a longer time is needed for learning to use the technology [31]—time that some stroke survivors do not want to waste [31]. Participants had limited exposure to technology for rehabilitation. Aging has stopped stroke survivors from making full use of the benefits of technology [31].

Unlike the younger generation that grew up with the internet, stroke survivors are not keen on going *online* [31]. In fact, security and safety of personal information were primary concerns of stroke survivors when talking about connecting social networking websites to home-based rehabilitation technology [31]. Integration of social networking negatively influences the potential acceptance of such rehabilitation programs [31]. Therapists emphasized that a system should be able to save individual settings and data of a stroke survivor [29].

### Theme 3: Decision-Making Process

#### Knowledge, Evidence-Based Practice, Advice, Time Investment, Safety Aspects and Regulations, Trust and Expected Usefulness, Independence and Self-Management, and Money.

The decision-making process for AT consists of factors important to both stroke survivors and their carers as well as HCPs. Stroke survivors are eager to function independently during ADL through self-management [16]. Stroke survivors expect that home-based technology would give them more independence in their rehabilitation program [27]. In addition, stroke survivors, carers, and their HCPs mentioned that an AT should be used independently at home [27,30], without the direct assistance and presence of an HCP (70%) [5]. Independent use of the AT is something that should be facilitated by the hardware and software design [16,29]. The design of the device in terms of safety, such as suitable solutions for emergency situations (back-drivable mechanism and quickly removable from the stroke survivor), electrical safety, and safety for the environment, plays a role in the decision-making process as well [19,27].

Some of the participants are actively engaged in the search for solutions to promote arm recovery [5,16], although there are many stroke survivors who have little to no exposure and knowledge about AT [16,31]. A majority of the HCPs, stroke survivors, and carers experience difficulties in accessing training and advice on AT, whereas stroke survivors and carers rely on the information given to them by HCPs. Ideally, they would like to seek advice from an HCP they know and trust [16]. However, stroke survivors feel that they receive too little information because HCPs lack knowledge and training about the availability of AT, HCPs are overworked, and because the therapists are reluctant to give information about devices that would not be state funded [16]. HCPs feel the tension about informing stroke survivors about the existence of a device, which may help, but which is not available from state-funded services [16]. HCPs prefer not to proactively inform stroke survivors about AT to prevent stroke survivors from purchasing an upper-limb AT for which insufficient research evidence is available [16]. For HCPs, scientific evidence is crucial [5,16,29], whereas stroke survivors and carers are less interested in the generic scientific evidence [5] and are more willing to accept risks [16]. Stroke survivors and carers point out that the evidence should be sought on a case-by-case basis because of the huge variety in the stroke population [16]. There is hope that AT could help stroke survivors to regain lost capabilities [28], and despite a potential lack of scientific evidence, HCPs believe that AT can enhance hands-on physiotherapy [27].

Although stroke survivors are willing to spend time and money on potential solutions [16], the decision-making process to invest in an AT largely depends on the financial commitment they have to make [31]. Concerns were raised by stroke survivors, carers, and HCPs about the current lack of financial support for AT and whether they will be cost-effective [5,16,27,31]. The amount of money HCPs, or their institution, would be willing to spend on an AT is less than US $10,000 for the majority (81%) of the respondents [30].

### Theme 4: Usability

#### Donning and Doffing, Setup, Initialization Time, Portable, Robustness, Instruction on Exercises, Comfort, Lightweight, Ease of Use, Compliant, Adjustment to Patient, Technical Support, and Maintenance

When a device lacks in usability, using it will be less pleasant, which can ultimately lead to device abandonment. As previously mentioned, independence and self-management are very important to stroke survivors. Usability factors that can contribute to independent and pleasant use of the device are (1) easy to setup [5,16,27-29,31], (2) simple to apply [16], (3) easy to don and doff without the aid of others [15,16,19], (4) quickly initialized [15,28,29,31], (5) comfortable to use and wear [5,15,19], (6) portable [16,27,28,30], and (7) lightweight [15,19]. A common generic theme mentioned by stroke survivors, carers, and HCPs in almost every paper is the ease of use of an AT [5,15,16,27-29,31]. This theme comprises simplicity [28,31], easily programmable [16], intuitive in terms of positioning, easy to operate [15], and short familiarization time [29] of an AT.

To be usable for both stroke survivors and HCPs, adjustment to the stroke survivor must be straightforward. An AT must comply with both left- and right-side affected stroke survivors [28]; concerns are expressed about complex adjustment between stroke survivors [16]. Both hardware and software should facilitate adaptation between stroke survivors, but it should also be adaptable to the stroke survivor’s progression over time [19,29].

For an AT to be used at home, stroke survivors and their HCPs want the device to be compact enough to fit in the home environment [27,28,30]. The AT must be deployable in a living room, kitchen, or bedroom [27] and should not hinder during ADL [19]. Moreover, stroke survivors and HCPs should be able to rely on the AT; therefore, it should be durable [5,29]. As there is a chance of an AT breaking down, it is preferred that access to engineers and to HCPs who have knowledge about the technology is available at any time [27].

### Theme 5: Applicability in Practice

#### Monitoring, Feedback, Wrongly Executed Movements, Fatigue and Overtraining, Adaptability, and Physical Comfort

Stroke survivors, carers, and HCPs acknowledge that ATs can potentially benefit functioning of stroke survivors by providing intensive therapy and a means of self-management [16]; however, factors influencing the implementation define the chances of user acceptance of AT in the long run. All respondents were of the view that ATs are efficient use of therapy time [5] and could be used to promote the usage of the hand and arm at home. Technology with the purpose of promoting hand and arm performance should first and foremost address the therapeutic principles mentioned in theme 1, that is, *promoting hand and arm performance*. Besides this, stroke survivors and HCPs want the possibility of an AT to be used unsupervised at home, which is why monitoring of their progression and provision of feedback are preferred. Among other reasons, monitoring and feedback are needed to halt or prevent wrongly executed movements, which can cause injury or inhibit recovery [29,30]. Compensatory movements are most likely to occur when fatigued, so an AT must monitor the state of fatigue of the stroke survivor [31]. The ability to monitor stroke survivor’s performance and quality of undertaken movements is seen as an important requirement to highlight possible problems [15,27,29]. Feedback not only plays a role for the HCP but also is key to support self-management [16]. Feedback on performance [15,16,28] and biofeedback were said to be of importance to stroke survivors and HCPs. However, stroke survivors do not necessarily wish for feedback from the system but rather prefer to receive feedback from the HCP [15].

Individual physical and cognitive impairments that limit the ability of a stroke survivor to perform tasks should be considered when applying a system in daily practice. HCPs are worried that different types of support are needed in ADL because of the individual impairments [15]; therefore, an AT must accommodate to the level of impairment and address movements that the stroke survivor needs to improve [28]. A modular system might not only fit into the individual needs of impairment level [15] but also technological familiarity [28]. Concerns are also expressed about the potential risk of harm such as secondary tissue changes, obstruction of blood vessels, sharp parts, and high forces that might cause injuries [5,19,28,30].

Besides adjustment between stroke survivors, an AT must be adaptable to the stroke survivor’s progression over time by adapting, for example, the level of difficulty [29], provided resistance and assistance [30,31], and the executed movements [28,30]. Automatic adaptation of task settings to account for the variation in impairment level is preferred as stroke survivors only want assist-as-needed: support only during (parts of) activities that need assistance [15,19].

### Relations Between Factors and Themes

**Figure 4:**
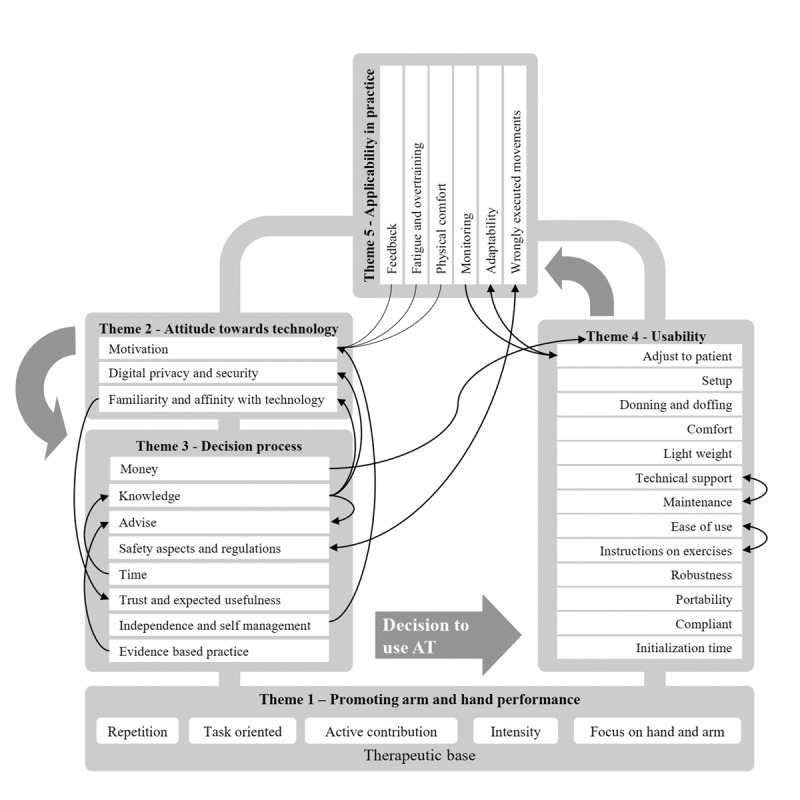
Factors and themes influence one another. Connecting lines indicate relationships between factors. AT: assistive technology.

The previous paragraphs discussed the factors within each of the 5 overarching themes. From the included studies, it is clear that the factors can affect one another, and there are also relations between the overarching themes. The main relations between factors and themes are mapped in Figure 4.

### Use Context of Assistive Technology

**Figure 5:**
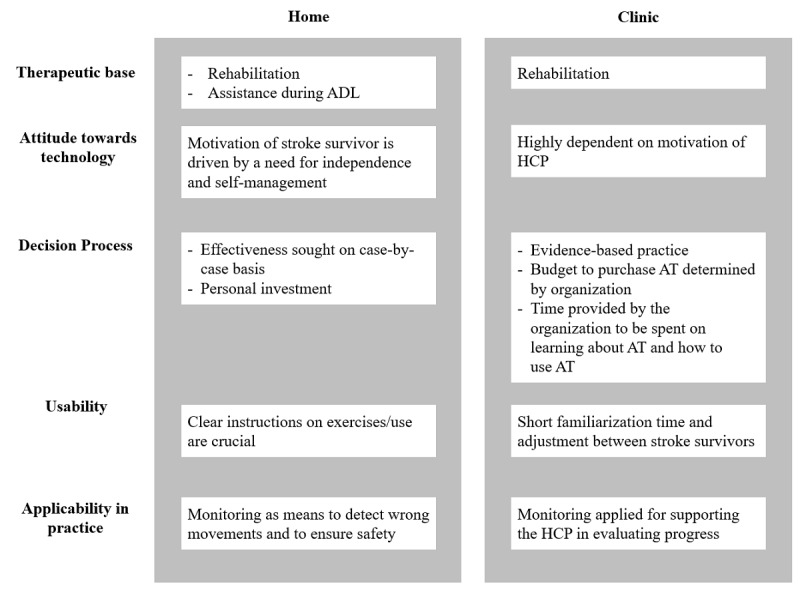
The importance and interpretation of the factors may differ depending on the use context of assistive technology. ADL: activities of daily living; AT: assistive technology; HCP: health care professional.

ATs are designed to be used either in the clinic or during daily life in a domestic situation. Although the definition of all themes and factors will differ to some extent between an AT used in the clinic or at home, the most pronounced differences are displayed in Figure 5.

## Discussion

### Principal Findings

This review comprehensively investigated user needs, preferences, and expectations that are expected to be associated with acceptance and adoption of AT for promotion of hand and arm performance after stroke. Through a meta-synthesis, 5 overarching themes were identified from literature. Factors relevant to stakeholders who may purchase or decide to use AT are covered in the following themes: (1) promotion of hand and arm performance, (2) attitude toward technology, (3) decision-making process, (4) usability, and (5) applicability in practice. Although separately presented by themes, the findings of this review highlighted the diversity and interdependence of the numerous factors influencing the chances of acceptance and adoption of AT, as illustrated in Figure 4.

### Interdependency of Themes and Factors

The potential of AT for the upper limb has been recognized by stroke survivors, carers, and HCPs [16]. Multiple stakeholders are directly or indirectly involved in the use of AT. Where stroke survivors and carers put more focus on self-management, HCPs put more focus on evidence-based practice. However, it is important to address the needs of every end user category during the design process [20] as involvement of both HCPs and stroke survivors will decrease the chance of discrepancy between expected and experienced usefulness. Unsatisfactory user interaction, or moreover, a lack of consideration of user needs, might lead to device abandonment [5,36].

Results from this systematic review suggested that adoption of AT depends on multiple organizational and psychosocial factors and can be influenced at any stage, ranging from attitude toward technology, to the practical applicability of AT designed to promote hand and arm performance after stroke. Previously, several general design criteria with a primary focus on usability have been developed [37]. The currently identified themes and underlying descriptive factors reflect many of those established design criteria. Moreover, several additional factors were identified in this review beyond those design criteria addressing predominantly usability, which are mainly represented by the themes *attitude toward technology* and *decision process*. Both themes affect the organizational process either by playing a facilitating role or by serving as a barrier. Besides that, factors such as age, gender, and voluntariness of use as described by the Unified Theory of Acceptance of Use of Technology influence the chances of adoption of technology [38].

To bring AT design to higher levels of user satisfaction and acceptance, the interdependency of user needs as revealed in this review must be considered in every stage of the design process. This means that addressing one particular aspect of the user perspective will not be sufficient to enhance user acceptance as, that aspect, for example, usability, is influenced by other aspects as well, for example, the budget available to purchase the AT (which is in turn dependent on the use context, for instance). Therefore, when designing AT to promote hemiparetic arm and hand function, the complete spectre of themes encompassing the user perspective, as identified in this review, should be addressed.

Motivation to use AT for upper limb after stroke is driven by the wish for independence and self-management. Therefore, use of AT should have substantial added value for the performance of task-oriented activities with the upper limb. In particular, activities that the stroke survivor would normally not be able to perform without assistance should be supported by AT. ATs are seen as efficient use of therapy time [5] and could be used to promote the usage of the hand and arm at home. However, before AT can be applied efficiently, the time required to (learn to) use AT plays a crucial role in the acceptance of AT for stroke survivors as well as HCPs. The time it takes for acquaintance is highly dependent on usability aspects such as donning and doffing, initialization time, and time needed to setup the device. Additionally, the practical applicability in terms of time needed to adjust the settings between or within stroke survivors affects the chance of acceptance. However, if an AT is effective in supporting self-management, stroke survivors are willing to spend time, and if necessary money, on it [16]. Naturally, their willingness is dependent on the financial commitment they have to make. Costs associated with AT, and a potential lack of funding, are seen as major factors influencing the decision on purchasing an AT. In terms of accessibility, concerns not only exist regarding purchasing the equipment and whether the time needed from staff can be billed at the insurance [29,39] but also with regard to informing stroke survivors about the existence of a device that may help but is not available from state-funded services [16].

Cost-effectiveness is seen as a determinant for the adoption of any new treatment [5]; it, however, does not automatically guarantee adoption into clinical practice or daily life [5,40]. Strength of scientific evidence has also been proposed to be an important factor influencing the translation of rehabilitation research into clinical practice, but there also appears to be a mismatch between the strength of the evidence and the clinical use of AT [5,41].

The decision-making process of HCPs to purchase or use an AT, or even inform stroke survivors about AT, is largely influenced by the level of knowledge about AT and the scientific evidence present. The decision-making process of stroke survivors is influenced by the HCPs as the primary source of information about AT is their HCP whom they trust. As only 25% of the devices have been tested in stroke [18], the clinical application and implementation remain low [39,42]. Currently, HCPs rely on their own experience with AT because of the absence of clear research evidence [5]. As proposed by Hughes et al [5], collaboration between clinical and developmental sites, health care providers, and the commercial sector would allow for a pragmatic approach for HCPs to learn about AT without awaiting publication, real dissemination, and reception of scientific evidence.

### Design Practice

Currently, the design of robotic technology for stroke rehabilitation tends to be technology-driven [22]. The focus on high-tech may jeopardize the consideration for (clinical) needs of the target population, which is a major reason why development can benefit from UCD methods. Unfortunately, manufacturers of medical devices in general can be hesitant in the involvement of users in the later stages of the design process because of perceived barriers in obtaining ethical approval and time constraints, among other reasons [43].

Cherry et al [44] reported on the perceived facilitators and barrier of stroke survivors after use of a hand telerehabilitation system for 3 months at home. Although many reported barriers and facilitators are in line with usability factors identified in this review, stroke survivors were able to point out the technical difficulties more specifically after actually using the device in their own homes. For example, unresponsiveness of the system that required rebooting, limited adhesiveness of the Velcro that was used, and incompatibility with existing furniture. New information about perceived facilitators and barriers as a result of prolonged use of a prototype or product highlights the importance of including user perspectives in the beginning of the design as well as later during evaluation of the prototype or product.

Developers should be aware that not only the prototype but the device itself can be evaluated with users. The instructions for use, commonly created in the wrap-up phase of development when all product details are known, can have great impact on usability. Quality of the user manual can be easily improved by giving several end users some assignments with the manual to determine whether the device can be successfully applied by following the instructions. In case of digital applications, it may be possible to collect user feedback after implementation to continue to improve the device through software updates, but developers need to seriously consider any privacy concerns users have, particularly in case of digital applications.

### Study Limitations

In this review, primary or secondary end users were not included during the sessions in which the overarching themes were defined. Instead, people who have experience in the design of assistive devices participated. Their backgrounds were diverse and with their different roles in device design, it was possible to combine the results into a complete framework that is useful to both developers of AT and those who evaluate or apply AT in practice. Inconsistent terminology about AT used among studies affected our ability to identify relevant studies. An iterative search strategy tailored to the databases was supplemented by scanning the reference lists of potentially relevant papers in an attempt to identify all relevant papers.

In addition, lack of distinction between AT used for therapeutic purposes and AT used during ADL in many studies made it difficult to design a framework for both purposes separately. Although the identified overarching themes are applicable in both situations, some factors may weigh heavier than others for either therapeutic or ADL purposes. For example, for a device that is to be used at home by only 1 stroke survivor, a low adjustment time is not as crucial as when the device is intended to be used by several stroke survivors on 1 day at the clinic. In this review, both focus groups and interviews and user survey studies were included in the meta-synthesis. Although the diversity in methods to elicit user perspectives might have influenced the results or its interpretation, the aim of this review was to include all relevant information on user perspectives about AT for the upper extremity after stroke. Valuable authentic information was retrieved from user survey studies, extending the development of factors and themes with unique data from a large(*r*) sample of potential users. It may be that the importance of factors varies between studies (or user-interaction methods), but weighing factors could not reliably be assigned in this review. Of the included studies, 2 studies had a methodological quality score below 5 [19,29]. Those studies particularly contained insufficient information about the recruitment strategy, data collection, relationship between researcher and participants, consideration of ethical issues, and provided an unclear statement of findings. Although rated low, those studies contained authentic information that contributed valuably to the comprehensive overview of themes related to user needs for AT for the upper limb as identified in this study. Another limitation is a potential selection bias in the reviewed studies where only participants who were already interested in the use of technology for the upper extremity were included in the study. This may have biased the views expressed by the participants in those studies. On the other hand, the various papers collectively included participants both with and without prior knowledge about and experience with AT.

### Future Work

The 5 themes as identified in this study are relevant to aid future AT developers in quickly determining essential user requirements as a first step of a UCD process. As stated before, the factors identified in this review have interdependency, and the importance of a factor may change depending on the use context. Therefore, all factors need to be considered within the specific use context for which an AT is being developed. However, the reviewed studies did not indicate if certain user needs were more important than others. Therefore, insufficient information was present to rank the importance of the factors or themes, but it would be highly relevant to assess the weights that should be attributed to the identified factors and themes in future research. After identification of the user requirements, design solutions can be created and developed [20]. The results gained from the focus groups, interviews, and questionnaires of the studies included in this review primarily reflect the expectations about AT use before actual usage of technology. The chance of actual use of a device is probably related predominantly to the experienced ease of use and perceived usefulness of the system [19,45], which cannot always be predicted beforehand. Therefore, subsequent evaluation of the newly designed AT in terms of a priori user preferences and corresponding user acceptance might give new and more specific insights into the (key) user preferences for an AT.

### Conclusions

This systematic review on user perspectives on AT identified several factors and themes that reflect user preferences for AT for the upper limb post stroke, before its development. The study identified barriers and enablers influencing the adoption of AT for the upper limb after stroke within the 5 overarching themes; (1) promoting hand and arm performance; (2) attitude toward technology; (3) decision process; (4) usability; and (5) practical applicability. Besides insight into relevant aspects for design of AT, this review showed that those aspects are highly interdependent. A potential purchaser of AT goes through a decision process. Prerequisite for entering the decision process is a sufficient positive attitude toward technology and the desire to increase independence and self-management of the stroke survivor. The stroke survivor and their carer(s) prefer to consult with a trusted HCP, who may or may not have experience with AT. By combining factors such as money, expected usefulness, and safety aspects, a decision can be reached to purchase AT. If AT incorporates therapeutic principles and can be used pleasantly in a time-efficient and safe manner, chances of acceptance increase. Time efficiency can be increased by usability factors such as setup time, clear and understandable instructions for use, easy donning or doffing, and adjustability. Features such as monitoring fatigue and detecting wrongly executed movements can contribute to safety. Depending on the use context, either at home for ADL purposes or for rehabilitation at a clinic, the importance of each factor may vary.

Due to this interdependency and a lack of weights attributed to the factors in the included studies, a ranking of most important themes could not be established within this review. Therefore, the current framework should be supplemented by future research evaluating the importance of the factors, while also considering differences in use contexts, such as clinical or domestic application of AT.

## Appendix

Multimedia Appendix 1 Search strategy.

## Abbreviations

ADL: activities of daily living
AT: assistive technology
CASP: Critical Appraisal Skills Program
HCP: health care professional
ICF: International Classification of Functioning, Disability and Health
UCD: user-centered design
