# Supplementary material for: Assistive Technology for the Upper Extremities After Stroke: Systematic Review of Users’ Needs
Source: JMIR Rehabil Assist Technol. 2018 Nov 29;5(2):e10510. doi: 10.2196/10510 (PMC6293243; doi:10.2196/10510)
Supplement: Multimedia Appendix 1 [file rehab_v5i2e10510_app1.pdf]

Search strategy for PubMed*

1. “Stroke” [MeSH Terms]
2. Stroke
3. CVA
4. Cerebrovascular accident
5. #1 OR #2 OR #3 OR #4

6. Self-help devices [Mesh Terms]
7. “Self-help”
8. “devices”
9. “self-help devices”
10. “help”
11. “Self” AND ”help ” AND “device”
12. Assistive
13. Rehabilitation [MeSH Terms] and Technology [MesH Terms]
14. “Technology”
15. #6 OR #7 OR #8 OR #9 OR #10 OR #11 OR #12 OR #13 OR #14

16. User

Combination of

17. #5 AND #15 AND # 16

*This strategy provided that basis for adjustment to other databases
